# Systematic Review of Treatment Failure and Clinical Relapses in Leishmaniasis from a Multifactorial Perspective: Clinical Aspects, Factors Associated with the Parasite and Host

**DOI:** 10.3390/tropicalmed8090430

**Published:** 2023-08-29

**Authors:** Gustavo de Almeida Santos, Juliana Mendes Sousa, Antônio Henrique Braga Martins de Aguiar, Karina Cristina Silva Torres, Ana Jessica Sousa Coelho, André Leite Ferreira, Mayara Ingrid Sousa Lima

**Affiliations:** 1 Postgraduate Program in Health and Environment, Center for Biological and Health Sciences, Federal University of Maranhão, São Luís 65080-805, Brazil; gustavo.as@discente.ufma.br; 2 Department of Biology, Center for Biological and Health Sciences, Federal University of Maranhão, São Luís 65080-805, Brazil; juliana.ms@icb.usp.br (J.M.S.); antonio.henrique@discente.ufma.br (A.H.B.M.d.A.); karina.cristina@discente.ufma.br (K.C.S.T.); ajs.coelho@discente.ufma.br (A.J.S.C.); leite.andre@discente.ufma.br (A.L.F.); 3 Postgraduate Program in Health Sciences, Center for Biological and Health Sciences, Federal University of Maranhão, São Luís 65080-805, Brazil

**Keywords:** visceral and cutaneous leishmaniasis, therapy failure, recurrence

## Abstract

Leishmaniasis is a disease caused by protozoa of the genus *Leishmania*. Treatment options are limited, and there are frequent cases of treatment failure and clinical relapse. To understand these phenomena better, a systematic review was conducted, considering studies published between 1990 and 2021 in Portuguese, English, and Spanish. The review included 64 articles divided into three categories. Case reports (26 articles) focused on treatment failure and clinical relapse in cutaneous leishmaniasis patients (47.6%), primarily affecting males (74%) and children (67%), regardless of the clinical manifestation. Experimental studies on the parasite (19 articles), particularly with *L. major* (25%), indicated that alterations in DNA and genic expression (44.82%) played a significant role in treatment failure and clinical relapse. Population data on the human host (19 articles) identified immunological characteristics as the most associated factor (36%) with treatment failure and clinical relapse. Each clinical manifestation of the disease presented specificities in these phenomena, suggesting a multifactorial nature. Additionally, the parasites were found to adapt to the drugs used in treatment. In summary, the systematic review revealed that treatment failure and clinical relapse in leishmaniasis are complex processes influenced by various factors, including host immunology and parasite adaptation.

## 1. Introduction

Leishmaniasis is a group of chronic infectious diseases caused by a group of protozoan parasites comprising more than 20 species of the genus *Leishmania*, which have different types of clinical manifestations, recognized by the World Health Organization (WHO) as cutaneous leishmaniasis (CL), mucocutaneous leishmaniasis (MCL), and visceral leishmaniasis (VL) [1,2].

VL symptoms include splenomegaly, irregular fever, anemia or pancytopenia, weight loss, and weakness occurring progressively over a period of weeks or even months [1]. CL is the most common form and causes skin lesions, mainly ulcers, on exposed parts of the body, and MCL leads to partial or total destruction of the mucous membranes of the nose, mouth, and throat [2].

Early diagnosis prevents severe clinical manifestations and reduces death, specifically in VL, which is fatal if left untreated in over 95% of cases. A diagnosis is made by combining clinical signs with parasitological or serological tests in VL; however, in CL and MCL, serological tests have limited value and clinical manifestation, with parasitological tests confirming the diagnosis [2]. Careful clinical evaluation and good parasitological examinations reduce diagnostic time and allow for early treatment with chemotherapy.

Chemotherapy is the main tool for controlling the parasite in the host; however, the available therapeutic set is quite limited, being currently restricted to two drug lines: antimonials (sodium stibogluconate (Pentostam^®^) and meglumine antimoniate (Glucantime^®^)) and non-antimonials (pentamidine, amphotericin B, paromomycin, and miltefosine). Azole derivatives (ketoconazole, itraconazole, and fluconazole) can be used, in addition to these, in the treatment of CL and MCL [3,4,5]. These drugs can be used individually or associated with each other (combined therapy) in all clinical manifestations (CL, MCL and VL), depending on the clinical condition of the patient and the judgment of the medical team.

The time of treatment is variable, depending on the type of clinical manifestation and geographical region. In general, Glucantime^®^ is administered for 28 to 30 days and amphotericin B is administered daily or every other day in 15 to 20 doses for VL, while miltefosine treatment is administered daily for 28 days for CL [2]. Treatment regimen (duration, dose, scheme) is associated with treatment failures (TFs) or clinical relapses (CRs).

Treatment failures and clinical relapses are not uncommon in leishmaniasis. TFs are characterized by cases where pharmacological therapy fails to reduce the infectious process and, consequently, does not lead to the cure of the disease. On the other hand, cases of a resurgence of clinical signs and symptoms after the end of treatment are referred to as CR [6].

However, the WHO presents conceptual variations for TF and CR in leishmaniasis, according to each region of occurrence of this disease in the world and the type of clinical manifestation, available in specific documents (see Refs. [7,8,9,10]). In addition, TF and drug resistance are often reported as synonyms in the literature, although they are different concepts. Drug resistance of parasites is just one of the factors that can lead to TF [11].

In this context, the present article aims to carry out a systematic review of TF and CR in the different clinical manifestations of leishmaniasis. We present, from a multifactorial perspective, the main clinical and epidemiological characteristics of the patients described in case reports with TF or CR, as well as a synthesis of the main findings in the literature with experimental studies that make it possible to explain the contributions of the parasite and of the host to these clinical manifestations.

## 2. Materials and Methods

### 2.1. The Literature Review Protocol

This study is a systematic review of the literature, whose protocol was submitted to the Prospective International Registry of Systematic Reviews (PROSPERO) under the following number: CRD42022320963 (https://www.crd.york.ac.uk/prospero/display_record.php?ID=CRD42022320963 (accessed on 25 April 2022)). We followed the guidelines of the Preferred Report of Systematic Reviews and Meta-Analysis (PRISMA) to conduct this review, as a guarantee of scientific rigor.

### 2.2. Data Sources and Research Strategies

**Figure 1:**
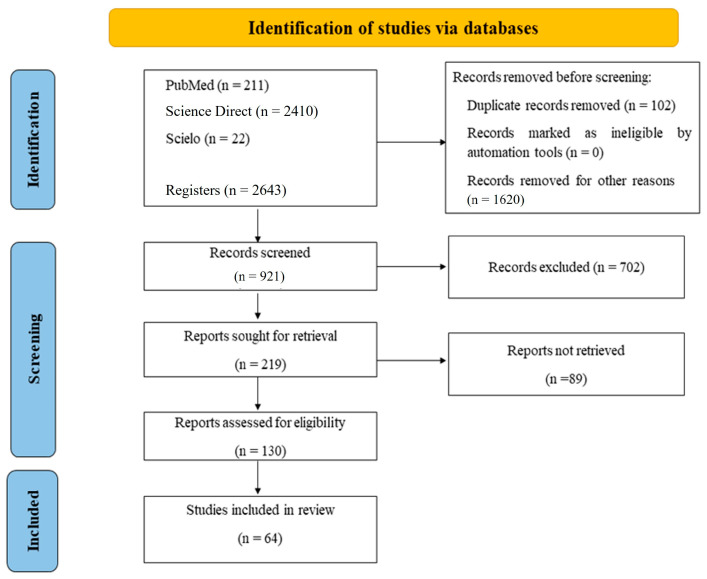
Flowchart of searches performed following the Preferred Reporting Items for Systematic Reviews and Meta-Analysis (PRISMA).

This review used articles published between the years 1990 and October 2021 that addressed the issues of CR and TF in leishmaniasis. The structured searches were carried out in PubMed, Science Direct, and Scielo databases. The following descriptors previously selected by the website Descriptors in Health Sciences (DeCS) access at: https://decs.bvsalud.org/ (accessed on 25 April 2022) were used: leishmaniasis, relapse, recurrence, recurrent, recrudescence, treatment failure, and therapeutic failure, as well as their equivalents in Portuguese and Spanish, used individually or in combination with the Boolean operators OR and AND. A summary of the selection of articles can be seen in Figure 1.

### 2.3. Inclusion and Exclusion Criteria

Population and experimental studies focusing on the human host, experimental studies focusing on the parasite, and case reports that addressed clinical recurrences and treatment failures in human patients, considering all clinical forms of the disease, were considered eligible. Languages considered were Portuguese, English, and Spanish, within the time frame between 1990 and 2021.

Studies with incomplete information, duplicated in the databases, in non-pre-established languages, and those with an approach focused on non-human hosts, as well as other reviews, were excluded from this analysis. We also conducted a search in the list of references of other reviews with the same approach as ours, for the inclusion of any additional articles that met the pre-established criteria.

### 2.4. Selection Process

After reading the titles and their respective abstracts, a blind selection was conducted by two reviewers using Mendeley software version 2.61.0, considering the inclusion criteria of this work. Abstracts that presented complete information were selected for full reading of the article to assess their adequacy in terms of the eligibility criteria. Independently, two reviewers read all selected works and in case of disagreement, a third reviewer assessed the studies.

### 2.5. Data Extraction

Data extracted from the articles were organized in Microsoft Excel tables according to the approach category of each study (case reports, experimental articles focused on the parasite, and population studies focused on the host). Some variables were observed for all categories of studies, which were as follows: year of publication; first author; origin of the article/place of occurrence; species of *Leishmania* under study; and the clinical form caused by said species. The selected variables of the case report studies specifically were as follows: sex and age of patients; therapeutic regimens employed; clinical outcomes; presence of immunosuppression and associated diseases (hypertension, diabetes mellitus, kidney and liver problems, among others) in patients; and the suggested mechanisms for failure/relapse by authors. For the experimental articles focused on the parasite, the variables specifically evaluated were as follows: experimental model used in study; use of reference strain and/or clinical isolate in experiments; execution of experiments using promastigote and/or amastigote forms; and drugs employed in tests and possible mechanisms suggested for parasites sensitivity alterations. Finally, for population studies focused on the host, the variables specifically evaluated were as follows: number of patients; drug(s) used; sex and age range; methodology employed in the study; presence of HIV coinfection in patients; and occurrence of and factors associated with TF and CR.

### 2.6. Analysis

For the interpretation and presentation of the results, we used the methodology of descriptive statistics to organize, summarize, and describe the important aspects of the main characteristics analyzed. Regarding the comparison of data, we described the main existing trends among the findings, such as similarities, differences, and particularities, depending on the approach used in each study.

## 3. Results and Discussion

This section may be divided by subheadings. It should provide a concise and precise description of the experimental results, their interpretation, as well as the experimental conclusions that can be drawn. The articles included in our study were categorized into three broad areas: case reports, experimental articles focused on the parasite, and population studies focused on the host. The first group presents results based on the description of clinical and epidemiological aspects of cases reported in different regions of the world, classified as CR or TF. Articles focusing on the parasite describe results based on tests of susceptibility of *Leishmania* to drugs *in vitro* and/or *in vivo*, as well as the use of molecular, genomic, and post-genomic approaches to identify the species or to understand the mechanisms related to drug resistance. Finally, the third group includes research with results that carried out epidemiological investigations from patient records, with a prospective and retrospective approach, as well as molecular and immunological laboratory tests.

Our survey encompassed the technological and methodological advances of the last 30 years regarding TFs and CRs in leishmaniasis, aiming to understand, from the most initial works that used the research methods available at the time to the most recent, the occurrence of TF and CR in leishmaniasis.

### 3.1. Case Reports

**Figure 2:**
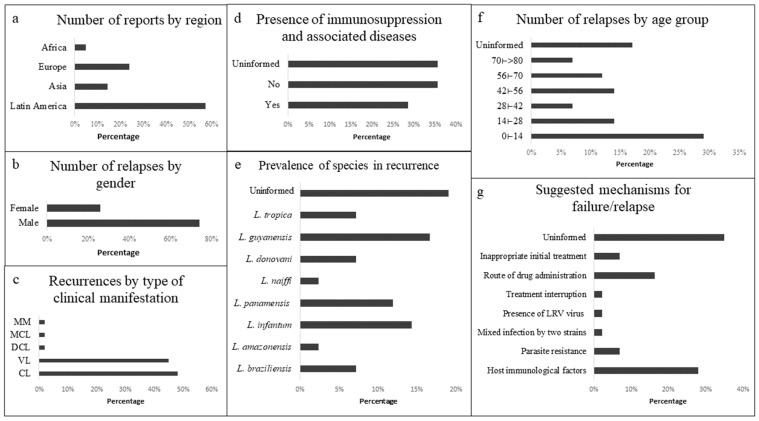
Chart with data collected from all articles classified as case reports. (**a**) The graph illustrates the number of case reports included in this study, segmented by geographical region. (**b**) The distribution of CR of leishmaniasis cases is presented, stratified by gender, as reported in the articles. (**c**) The graph displays the main types of clinical presentations of leishmaniasis that experienced clinical recurrences in the analyzed articles. (**d**) It demonstrates the presence of immunosuppression and associated diseases identified in the research participants. (**e**) The graph highlights the prevalence of different species of *Leishmania* spp. associated with CR, based on the species studied in each article. (**f**) It presents the relationship between CR and age groups, providing an overview of recurrence incidence across different age ranges. (**g**) The graph summarizes the mechanisms pointed out by the authors of the articles as responsible for TF/CR. CR: clinical relapse; TF: treatment failure; CL: cutaneous leishmaniasis; VL: visceral leishmaniasis; DCL: diffuse cutaneous leishmaniasis; MCL: mucocutaneous leishmaniasis; MM: multiple manifestations.

After applying the previously established exclusion criteria, we selected 26 works categorized as case reports, whose information can be seen in Table S1 (See Refs. [12,13,14,15,16,17,18,19,20,21,22,23,24,25,26,27,28,29,30,31,32,33,34,35,36,37]). The data were summarized by patient (42 in total) and their respective clinical and treatment histories. Latin America led among the reported places with the highest number of cases described (Figure 2a), with Brazil, Colombia, and French Guiana being the countries with the highest number of relapsed patients (seven each). In Brazil, the region with the highest occurrence was the Northeast; in both Guyana and Colombia, regions included the Amazon Forest area. According to the WHO’s classification of CL and VL endemicity status around the world, in 2020, all countries present in the reports described some degree of endemicity or had previously reported cases of CL and VL in their territories [38,39].

The clinical form with the highest number of treatment failures/clinical relapses reported was the cutaneous form, with 47.6% of patients affected (Figure 2c). These data are quite consistent since, according to the WHO, CL is the most common presentation of leishmaniasis [2]. However, the visceral clinical manifestation had a total of 45.2% of the patients with treatment failures/clinical relapses, thus not showing much difference in quantitative terms. Manifestations such as “Diffuse Cutaneous”, “Mucocutaneous”, and multiple manifestations (more than one clinical form present) were recurrent for a single patient each. In agreement with these data, the species identified in the largest number of reports were *L. (V.) guyanensis* (18.9%), *L. (V.) panamensis* (13.5%), both causing CL, and *L. (L.) infantum* (16.2%), responsible for the visceral form of the disease. Despite these data, most authors did not identify the species of *Leishmania* present in their patients (21.6%).

Regarding the sex of individuals affected with relapses, the male population displayed the most involvement, with 74% of patients being male and only 26% being female (Figure 2b). The trend in male prevalence in the occurrence of leishmaniasis also occurs at a global level: according to the WHO’s Global Surveillance of leishmaniasis (2019–2020) report, the distribution of the incidence of VL cases by gender in high-income countries showed that in 2020, 58% of cases were male patients and 42% were female patients. As for CL, in 2018, 47% of cases were female and 52% were male [40].

An interesting fact brought up by the aforementioned report is that some regions and countries present an overrepresentation of male patients, such as the Americas region, which only had 30% of female patients with CL in 2018, and the Brazilian country, which presented 89% of male VL patients in 2020 [40]. These differences may be attributed to social and behavioral factors (such as greater male exposure to areas of risk of infection and lower demand for health services), or biological factors directly associated with sex [41]. Some authors discuss that the prevalence of VL cases in the male population of the Indian subcontinent is not only due to sociocultural factors, observing that biological differences between the sexes play a role in the pathogenesis of the disease [42].

The age group with the highest reported number of occurrences of treatment failures/clinical relapses was children (0 to 14 years), with the visceral form in this group being the most common, representing 67% of cases (Figure 2c). Age as a risk factor for the occurrence of relapses has already been reported by several authors; age was pointed out as an important risk factor for VL and correlates childhood with a 5 to 10 times greater possibility of poor response to treatments compared to adulthood [43]. Similar findings were reported in a study with Colombian CL patients, which showed a low success rate of treatment with meglumine antimoniate in patients younger than 8 years old [44]. In a prospective study in health centers of India and Nepal, the clinical outcome of 1016 VL patients treated with miltefosine was evaluated, and the occurrence of relapses was observed as two to three times more frequent in the group of 0 to 15 years when compared to adult patient groups [45]. These results highlight the importance of conducting appropriate clinical trials for children since it is widely known that they have immature immune systems, and it is necessary to determine the safety and efficacy of drug exposure for these patients.

The presence of immunosuppressive states and associated diseases in patients was reported for 28.6% of individuals. About 35.7% of the patients were already indicated by the authors as immunocompetent, and the same number of individuals had no information presented about their immunological status. This is a high percentage and a worrying fact, considering that in practically all the reports of immunosuppressed patients, the host’s immunological factors were identified as responsible or potentially relevant in the occurrence of treatment failures/clinical relapses. However, for another 35% of patients, the authors did not hypothesize or suggest mechanisms and factors that could have led to their patients’ non-response to the treatment initially administered. These data represent, to a certain extent, an expected result, since the “Case Report” publication format does not require a discussion of the mechanisms involved in the reported phenomenon, but rather that it addresses the casuistry of relapse.

The immunological status of the patient has already been pointed out in other studies with our approach as a major determinant of the occurrence of relapses in the treatment for VL. In a recent study, it was demonstrated, in a cohort of 98 VL patients from Ethiopia, that relapse in VL/HIV patients is due to the persistence of parasites after a clinically successful treatment which, in the absence of fully functional host immunity, are capable of re-establishing themselves and causing repeated relapses [46]. A clinical cure in CL was also previously reported in the literature as rarely associated with a sterile cure, with relapse being directly linked to the persistence of parasites in the scars [47].

In addition to the absence of a sterile cure, several clinical characteristics and drug-related factors were highlighted before in the literature as determinants of the occurrence of TF [44,47]. In a cohort study conducted with 230 Colombian patients with CL, age, presence of regional lymphadenopathy, duration of infection, and poor adherence to treatment were highly associated with TFs [44]. In a study with 338 Iranian patients with CL, a positive correlation between the occurrence of treatment failures/clinical relapses and different demographic, clinical, and environmental risk determinants was found [48].

Factors related to the therapeutic regimen used were highlighted as relevant in 26% of the cases (17% on the route of administration used, 7% on inadequate treatment and 2% on interruption of treatment) (Figure 2g). It was not possible to visualize a pattern in treatments and possible relationships between drugs and a higher incidence of relapses: 52% of patients relapsed after using a single drug and 48% relapsed after using two or more unsuccessful regimens. The drugs most used worldwide in the treatment of leishmaniasis (Glucantime^®^-meglumine antimoniate and amphotericin B) were also the most frequently administered to patients with relapses. Patients in the studies evaluated in the present survey, treated with Glucantime^®^ (alone or in combination), had failure in 45% of cases, while those treated with amphotericin B (alone or in combination) represented 43% failure. These extremely close percentages show the low correlation between the choice of treatments and the chance of initial therapeutic success. Thus, it is possible to perceive that leishmaniasis has a low therapeutic range at the global level and the combination of therapies with pre-existing drugs does not guarantee success.

Intrinsic factors of the parasite (such as strain resistance, presence of *Leishmania* RNA virus (LRV), and infection with more than one strain) were associated with treatment failures/clinical relapses by only 11% of authors. It is important to highlight, once again, that the format of the works analyzed in this topic was “case report”, in which the focus of evaluation is on the patients and not on their etiological agents. The following topic discusses in more detail articles that produced experimental data about parasites of the genus *Leishmania* and their possible relationships with the TFs with conventional drugs.

### 3.2. Experimental Data Focusing on the Parasite

We included 19 articles in this category, described in Table 1 (Refs. [49,50,51,52,53,54,55,56,57,58,59,60,61,62,63,64,65,66,67]), which address several aspects that seek to explain the influence of the parasite in episodes of TF/CR. Of the articles included in this category, approximately 72.7% carried out research with species that cause CL, including: *L. peruviana*; *L. guyanensis*; *L. braziliensis*; *L. lainsoni*; *L. mexicana*; *L. killicki*; *L. amazonensis*; *L. panamensis*; *L. tropica*; and especially *L. major*, which corresponded to 25% of the studied TF/CR species in CL. The studies that used the species causing VL were equivalent to 27.2% and were carried out with the two species listed in the literature as responsible for the visceral manifestation of the disease: *L. (L.) infantum* and *L. (L.) donovani*. There was a higher prevalence of the second species, which corresponded to 77.7% of TF/CR research in VL. It is important to consider that studies with the species that cause CL were more comprehensive, addressing more than one species at the same time (50.0% of them); however, the studies with VL addressed only one of the two species. It was still possible to find two studies that compared CL and VL species simultaneously. According to the WHO, it is estimated that 50,000 to 90,000 new cases of VL occur annually worldwide, while in CL, this estimate is 600,000 to 1 million new cases annually worldwide [2]. In this sense, it is possible to understand why there are more cases reported in the literature and clinical/laboratory studies with CL, since there are more than 20 species known to cause leishmaniasis and only 2 of these are scientifically identified as responsible for VL in the world. The others are classified as etiological agents of CL and its derivations, with significant differences being found between the species. This is shown in a study that pointed out a difference in the sensitivity of *Leishmania* species to treatment with SbV in patients with CL, thus showing that the species and geographic region where the infection occurred can affect the effectiveness of treatments [49]. Among the drugs used, antimonials were predominant, reported by 52.6% of the studies, in which 90% of the antimonials were used in the pentavalent form of the drug (SbV) and only 10% in the trivalent form (SbIII), with commercial antimonials being the most used, including sodium stibogluconate (Pentostam^®^) and meglumine antimoniate (Glucantime^®^). Pointed out as one of the alternatives in the treatment of leishmaniasis, miltefosine was the second most used drug in the studies (26.3%) to evaluate the mechanisms of the parasite against the action of the drug. Research was also carried out with drugs not used in the treatment of leishmaniasis, such as glibenclamide, intended for the treatment of diabetes *mellitus*, but which may have a leishmanicidal effect (15.8%). Amphotericin B was addressed by 5.2% of the articles, being the least used drug in the TF/CR studies. It is important to emphasize that only articles that worked with commercial drugs were used in our study, not including drugs/compounds in the laboratory validation phase or studies with bioprospecting for new products. The wide use of antimonials in these studies is justified, as these drugs have been recommended for more than seven decades for the treatment of VL with a cure rate >90% at the global level and for the treatment of CL with a variation between 77–90% of cure rate in the world, being the first line of treatment in most countries, but presenting problems such as resistance in countries like India and Nepal, as pointed out by the WHO [68]. The majority (73.6%) of the studies used the two forms of presentation of *Leishmania* (promastigotes and intracellular amastigotes) in their investigations. Studies with promastigote forms were used in this context to perform *in vitro* resistance induction, analysis of parasite sensitivity to drugs, and gene assays. With intracellular amastigotes, as demonstrated before, it is possible to better understand the behavior of the parasite in mammalian cells through sensitivity tests, evaluation of alterations in the mediation of immune responses, alterations in biochemical pathways, functional assays, among others [53,60,63].

The experimental models used in the studies were classified into four categories: (1) *in vitro*/*in vivo* sensitivity assays, that correspond to 27.9% of the methodologies and encompass experiments that aim to evaluate phenotypes of sensitivity and drug resistance in parasites; (2) functional assays, performed by 11.62% of the studies and encompassing methods related to the understanding of biochemical and metabolic pathways, as well as protein interactions in the intracellular environment; (3) gene assays, representing 30.23% of the methods and including techniques used to evaluate mutations, alterations in chromosomal copy numbers, gene overexpression, presence of SNPs, and other alterations at the genomic level; and (4) identification/molecular characterization of the parasite, comprising 30.23% of the studies, using mainly PCR-RFLP or sequencing to identify the species of the parasites.

Regarding the possible mechanisms for alteration of sensitivity in the parasite, which aim to try to explain which means and factors in the parasite are related to TF/CR, the categorization was as follows: factors associated with DNA alterations or gene expression level (44.8%); alterations in biochemical pathways (20.6%); and factors associated with virulence and alterations in the rate of metacyclogenesis (10.3%). Natural interspecific variation, alteration of immune response mediation, presence of LRV virus infection, and stage-specific variation of the response were equivalent to 3.44% in each of the groups.

The first descriptions of the possible mechanisms of action of some of the drugs are classically addressed in the literature and were consolidated a few years ago. Antimony acts on different pathways that affect the parasite, presenting a variation in the stage-specific response, in which the resistance to sodium stibogluconate was already observed in the promastigote form of *L. donovani*, while the amastigote forms of the parasite were observed to be more sensitive to drug action [65]. There is also the presentation of a natural interspecific variation, analyzed previously, in which it is possible to observe that different species of *Leishmania* spp. have different responses to pharmacological therapy, and the type of species may influence the outcome of the treatment; for example, the chances of occurrence of TF, demonstrated by the authors, when they highlight that TF was 3.5 times more frequent in patients infected with *L. (V.) braziliensis* compared to those infected with *L. (V.) guyanensis* [49].

Regarding the factors associated with DNA alterations or at a gene expression level, going by a chronological order of the findings, we were able to observe that the first results already highlighted that resistant strains of *L. mexicana* exhibited an increase in the expression of P-glycoprotein [56], and that a gene with a locus on chromosome 9 in *L. major* isolates conferred resistance to antimony [57]. The expression of ABC (ATP-binding cassette) organic anion transporters by *Leishmania (L.) amazonensis* is associated with Glibenclamide resistance [52], which was also found in antimony-resistant *L. amazonensis* with increased ABC MRPA (multidrug-resistance protein A) gene expression and high thiol levels [54]. In a study with amphotericin B, differences in the expression patterns of S-adenosyl-l methionine:C-24-Δ-sterol methyltransferase transcripts and the absence of ergosterol, substituted by cholesterol 5,7,24-trien-3β-ol were observed [63]. In addition, changes in the level of MDR1 (multidrug-resistance 1) gene expression indicate a decrease in the affinity of *L. donovani* with the drug. Other authors pointed out an overexpression of resistance genes in strains of *L. (L.) infantum*, namely 14-3-3 (14-3-3 protein), P299 (protein 299), MRPA, GSH1 (γ-glutamylcysteine synthetase), TRPER (tryparedoxin peroxidase), AQP1 (aquaglyceroporin), and H4 (histone 4), as well as an overexpression of similar genes (14-3-3, P299, AQP1, and H4) in *L. major* isolates [66]. Studies also identified a relationship between MSL (miltefosine sensitivity *Locus*) deletions and sensitivity of *L. (L.) infantum* to treatment with miltefosine, correlating with clinical cases that presented CRs [67]. More recently, it was observed for *L. major* that the partial or total deletion of the Ros3 gene may be involved in the resistance of the parasite to miltefosine [53]. Thus, the data demonstrate that molecular factors, possibly associated with the alteration in the sensitivity of the parasites, are multigenic and have multiple response pathways involved, in addition to varying between species, since the same drug may be related to different manifestations of the disease-resistance phenotype in different species. In other words, it is not possible, from the genetic perspective, to carry out an individualized approach to understand the phenomenon of resistance and to correlate it with CR/TF, considering the expression of multiple genes involved with different response pathways for different drugs.

As for changes in biochemical pathways, it was initially observed for *L. mexicana* that resistant strains showed a decrease in the activity of key functional enzymes, such as acid phosphatase and pyruvate kinase [25,56]. It was also highlighted that protein phosphorylation could play a role in the signal transduction pathway in the parasite after drug exposure [64]. The action of butionine sulfoximine in the process of sensitization of the parasite was also demonstrated, as this is an inhibitor of γ-glutamylcysteine synthetase, being related to resistance to Glucantime^®^ [62]. Resistant *L. donovani* parasites have a more fluid cell membrane, enabled by the upregulated tryparedoxin cascade and a lower intracellular thiol level [63]. Also, in resistant strains of *L. donovani*, there was a reduction in miltefosine uptake, a decrease in ROS accumulation, and an increase in intracellular thiol content [60]. Therefore, it is possible to understand some of the changes that merit further studies, as they can be characterized as predictive of adaptive responses of each species in relation to certain drugs.

Regarding factors associated with virulence and increased metacyclogenesis of *Leishmania* spp., one study reports the high capacity of resistant strains of *L. donovani* to cause infection in vivo, with a heterogeneous profile of infection and a parasite load several times higher in relation to susceptible strains [58]. This was also verified by other authors who reported the highest fitness traits found in pentavalent antimony-resistant *L. donovani* strains, which have a greater capacity for metacyclogenesis [59]. Other studies found an increased capacity for infectivity and metacyclogenesis with this same species, which was also highlighted by other authors who state that miltefosine-resistant *L. major* promastigotes exhibit increased metacyclogenesis [57,60]. Therefore, it is possible to understand the process of adaptation of the *Leishmania* species that cause VL and CL, with presentations of parasites that are fitter, resistant to drugs, more virulent, and with greater infectivity, which may be an even bigger problem in the future, considering the restricted spectrum of drugs that are proven to be effective in the treatment of these diseases and can be used in humans without complications due to side effects.

Furthermore, the discovery of the presence of the LRV in *Leishmania* raised the question of whether it could influence the sensitivity of the parasite to drugs. However, a surveyed study revealed that there was no direct association between the presence of the LRV2 and the response to Glucantime^®^ [51]. Nevertheless, studies are still being carried out to better understand the impact of the presence of this virus on the development and treatment of the disease. In addition to the parasite aspects, it is also important to consider the factors associated with the host, given that the host’s immune system has a proven relationship in the involvement and treatment of the disease [69].

### 3.3. Population Data with a Focus on the Host

Host-focused articles were further divided into descriptive and analytical observational studies and experimental clinical trial-type work. We selected 19 articles in total, out of which 33% carried out immunological investigations in the hosts (immune cell count and/or cytokine dosage), 26% developed retrospective cohort studies, and 19% were prospective cohort studies. Two case series studies, a case-control study, a cross-sectional study, and an ecological epidemiological study were also analyzed. The mean number of patients in the studies was approximately 237, ranging from 4 to 1761 patients. All data are available and can be viewed in Table 2 (see Refs. [45,70,71,72,73,74,75,76,77,78,79,80,81,82,83,84,85,86,87]).

The percentage of articles that pointed out patients classified as relapsing was 57%, while for TF, it was 37%. Only two studies (11%) identified, among the investigated populations, both relapsed individuals and those with TF; these concepts were treated as different, without one overlapping the other. In contrast, among the 19 studies, 3 openly considered CR as a type of TF. Only one author used the term “unresponsive” explicitly as a type of TF [86]. We emphasize here that these data were quantified strictly based on what the authors of each article declared as TF, non-response, and/or CR. These concepts were divergent between the articles, with adaptations to the terminology defined by the WHO, especially regarding the frequency of the reappearance of symptoms.

Some authors followed the concept defined by the WHO; however, they adapted the time for reappearance of symptoms to 6–12 months or simply 12 months after the end of treatment [45,79]. In another study, the authors extended this period to 1 to 24 months after completing the initial therapy [77]. In addition to the variety of periods adopted, some authors considered other criteria for confirming the relapse episode. In one study, the authors did not establish a time interval for recrudescence but determined the visualization of parasites in spleen or bone marrow aspirates as necessary for the classification of relapse with the reappearance of symptoms [81]. Meanwhile, other authors linked the diagnosis of CR to the need for biological confirmation of the existence of parasites in a smear or culture examination of medullary or peripheral blood, with at least three of the following clinical criteria: intermittent fever, asthenia, weight loss, sweating, hepatomegaly, splenomegaly, respiratory, and gastrointestinal symptoms. In addition, they distinguished the concept of biological relapse, which corresponds to the transient recirculation of parasites detected by the PCR technique, and determined a threshold of parasitemia of 10 parasites/mL for the occurrence of CR [76].

When it comes to studies involving patients with cutaneous manifestations, definitions for TF and CR were even more diverse. Some authors classified TF in the first month after the last dose of the drug used, while others adopted the criterion of the persistence of lesions within 6 weeks after treatment [73,75,87]. TF was also considered incomplete re-epithelialization and/or presence of induration, raised edges, or redness in any lesion after day 90 and framed the emergence of new lesions and relapse as a type of TF [71]. However, other authors considered relapse based on the return of patients to the doctor’s office within 1 year after the initial diagnosis was made [85]. In another study, they did not determine a period for classification of CR, but they established that patients who had re-emergence of initially healed lesions after the end of treatment or that had plaques with satellite papules were classified as relapsing [86].

Out of the 19 articles, 10 evaluated patients with VL (and out of these, 6 with coinfected patients), 8 with CL, and 1 with MCL. Only one of the articles involving VL patients investigated the occurrence of post-kala-azar dermal leishmaniasis (PKDL). It is noteworthy that the vast majority of investigations with individuals presenting clinical manifestations of the skin were in Latin American countries, such as Brazil, French Guiana, Colombia, Peru, and Bolivia; meanwhile, populations of individuals with VL were studied mostly in Southeast Asian and African countries, such as India, Nepal, Sudan, and Ethiopia. According to the WHO, multiple circulation of different species of *Leishmania*, variations in transmission cycles, clinical manifestations, and response to therapy make the epidemiology of CL in the Americas a very complex phenomenon, while the main form of disease manifestation in the region and outbreaks of VL are common in East Africa and Southeast Asia [2].

Regarding sex predominance in the populations studied, 84% of the studies presented a majority in male involvement. As previously discussed in the topic “Case Reports”, the prevalence of male patients occurred for both VL and CL at the global level, and these results agree with what is observed in Figure 2b regarding case reports. Only one article involved more female patients than male patients in its investigation [59]. Three studies did not specify the number of male patients and female patients in the sample. The total percentages of men and women in all populations with described sex discrimination were 65% and 35%, respectively. In addition to social and epidemiological factors, some authors argued that experimental evidence and infections in humans suggest the existence of biological predispositions that justify the existence of differences related to the sex of individuals and their respective immune responses and manifestation symptoms in infections by *Leishmania* spp. [88]. However, the fact that more males develop the clinical manifestations of leishmaniasis needs to be better assessed and investigated, considering factors that may or may not be related to this phenomenon, such as differences in metabolism and/or hormones released between hosts of different sexes and possible differences in food preference of the hematophagous vector, among others.

Coinfection with the HIV virus was a variable present in 32% of the studies, all of which with patients presenting visceral clinical manifestations. This was an expected finding, since it is extensively reported in the literature that HIV infection increases the risk of developing VL; this, in turn, promotes the development of AIDS-defining conditions [40]. In studies with CL and MCL patients, the coinfection variable was not present. In 37% of the studies, it was not reported whether there was VL/HIV coinfection among the individuals in the study. According to the WHO, VL/HIV coinfected patients with a CD4+ count <200 cells/μL normally suffer from progressively frequent CRs, until they become unresponsive to all drugs used [89].

Six types of risk factors for TF/CR were identified by the authors. The immunological characteristics of the host were highlighted among 36% of the factors, the association being more frequently made as a risk factor among the analyzed studies. These data were consistent with what was previously found in the topic “Case Reports”, in which 25% of the case reports had TF/CR associated with the immunology of the patients (Figure 2g). It is worth highlighting that the average age of the populations studied was mostly adults, with only one study with a significant presence of patients in the child age group [87]. It is understood that, in numerical terms, the adult population is more representative of general cases of leishmaniasis: in 2020, 58% of the population affected by VL in 10 of the 14 high-burden countries were aged 15 years or older, while in 2018, 55% of the population affected by CL fell into this same age group [40]. However, we emphasize here the need for further studies specifically aimed at the child age group, given that children have immature immune systems, which may be associated with clinical outcomes characterized by TF.

Clinical host variables were associated with TF/CR by 29% of the authors. The therapeutic regimen was pointed out by 21% of the authors as a risk factor and the route of drug administration by 7%. Only one study (4%) highlighted the importance of LRV1, and one study showed ecological epidemiological factors as determinants.

The WHO points out that the risk factors for CR are as follows: non-performance of antiretroviral treatment; low CD4+ cell count; previous episodes of VL; and failure to obtain clinical or parasitological cure during the first episode, without secondary prophylaxis [87]. As the WHO defines CR as a form of TF, it is to be understood that these risk factors are also true for TF. The first two risk factors highlighted by the WHO are directly related to the findings in this research, where the majority (36%) of the authors pointed to host immunological characteristics as factors associated with the development of TF/CR, such as lower increases in CD4+ cell count, increasing the chances of CR, and in some cases, successful antiretroviral therapy in patients coinfected with VL/HIV, which may not be enough to control the disease [84]. According to some authors, female sex was considered a risk factor for the development of CR in patients with VL [83]. In another study, a presentation of high tissue levels of T CD8+ and NK cells, a low number of macrophages, and a high proportion of IFN-γ/IL-10-producing cells in a patient afflicted with mucosal leishmaniasis is highlighted as important for TF/CR; however, other authors consider that the level of activation of the Th1 response is greater in CL patients who had therapeutic failure to antimony than in patients who responded successfully to treatment [73,74]. The results of two other studies conclude that a young age, given the immaturity of the immune system, may compromise the final outcome after treatment [45,81]; in addition, male sex was associated with an increased risk of VL recurrence after treatment with miltefosine, as they believe that a failure to maintain the T-cell-dependent immune response is involved in this process. In another case, the significant increase in TGFβ is associated with TF in patients with VL treated with sodium stibogluconate [78]. Some studies highlighted that the absence of antiretroviral treatment and the T CD4+ cell count below 100 cells at the time of the first infection, as well as the maintenance of T CD4+ cell values below 100 in people affected with VL, were described by the authors as factors of risk for relapses and failures [71,84]. Other authors associated CR with HIV coinfection, the presence of lower limb edema, low platelet count on admission, and secondary pneumonia in people with the visceral manifestation of the disease [77].

Among the clinical factors associated with TF/CR described by the authors (29%), previous treatment for leishmaniasis, three or more lesions, irregular treatment, and weight greater than 68 kg were associated with TF in CL patients treated with meglumine antimoniate, as reported previously in the literature [70]. Some authors highlighted that risk factors for TF are disease duration <5 weeks, additional injury, and *L. (V.) peruviana* and *L. (V.) braziliensis* infection in patients with CL treated with sodium stibogluconate [72]. The presence of regional lymphadenopathy, disease duration ≤1 month, and low adherence to treatment (<90%) were also previously associated with TF [71]. It was observed in another study that CR was associated with HIV coinfection, the presence of lower limb edema, low platelet count on admission, and secondary pneumonia [77]. Moreover, other authors associate epidemiological risk factors for TF, such as a stay <72 months in the area of acquisition of the disease [72].

Regarding the therapeutic regimen factors (21%) and drug administration route (7%) associated with TF/CR, some authors conclude that the use of miltefosine as a monotherapy is not sufficient to cure relapsing VL in HIV-1-controlled infected patients [82]. Also, treatment with pentavalent antimony was associated with TF [71]. Another study evaluated cases of VL patients treated with liposomal amphotericin B (AmBisome^®^) and miltefosine in monotherapy courses who had multiple episodes of TF/CR [74]. Similar data were found by another study in which the incidence density of relapse was higher in patients with VL treated with amphotericin B and miltefosine [81]. Irregular and/or non-continuous recommended treatment were related to the development of TF/CR, just as treatment with AmBisome^®^ was also previously associated with a higher risk of developing CR [81,86].

When approached about the relationship between the route of drug administration and the emergence of TF/CR, a previous study stated that the administration of an intravenous regimen of sodium stibogluconate for patients with CL caused by *L. braziliensis* and *L. guyanensis* may be correlated with TF, with the intramuscular route being more effective and presenting decreasing hepatotoxicity [77]. However, other authors describe that the use of intramuscular pentamidine in the treatment of CL by *Leishmania guyanensis* is associated with more TFs than intravenous pentamidine isethionate [87]. Thus, it is considered that the use of monotherapy in cases of leishmaniasis may enter a long replacement process, given the higher frequency of TF/CR in this method and the possibility of using drugs concomitantly. Nevertheless, regimens with the use of more than one drug need to be well evaluated, considering the toxic effects that these drugs can have on the body. Thus, the real impact that the drug administration route had on the final outcome of the clinical picture of leishmaniasis is still unclear.

Interestingly, we found two studies that show that patients infected with *L. (V.) guyanensis* have a lower association between TF and pentavalent antimonials than patients infected with other species, such as *L. (V.) peruviana* and *L. (V.) braziliensis* [49,83]. However, other authors also studied patients infected with these species and the results were completely different, showing a high rate of TF in patients infected with *L. (V.) guyanensis* and treated with meglumine antimoniate, reinforcing the argument for the difference in response between species to the same drug, in addition to differences in the response of the same species in distinct regions [90].

The species *L. (V.) guyanensis* was also highlighted in studies that evaluated the presence and influence of LRV on the clinical outcome of patients, correlated to all studies found in the topics “Case Reports”, “Experimental data focusing on the parasite”, and “Host-focused population data” on LRV, in which the results were conflicting, showing a relationship between this species with TF and CR in immunocompetent patients treated with pentamidine and Glucantime^®^ in France, while in French Guiana and mainland France, opposite results were found in patients without HIV and treated with pentamidine [81,87]. In Brazil, a study hypothesized that the presence of LRV1 contributed to increased parasite virulence in immunocompetent patients infected with *L. (V.) naiffi* and treated with pentamidine; however, in Iran, some authors reported that the presence of LRV2 in *L. major* did not show significant differences in the response to Glucantime^®^ [25,50].

Many studies in the literature have previously found an influence of LRV infection on the virulence of parasites and the risk of development and worsening of mucosal lesions in patients with CL [91,92]. However, it is understood that the influence of LRV on the clinical outcome of patients has not yet been elucidated, and there is no consensus on its impact on the occurrence of TF and CR. No studies were found that addressed the presence of LRV in VL, which was an expected result, since the presence of the LRV1 is reported only in South American parasites, which cause cutaneous manifestations, of the subgenus *L. (Viannia)* [93]. However, the only occurrence of LRV outside the *Viannia* subgenus was described, which occurred in the *L. (Leishmania) major* strain (MHOM/SU/73/5-ASKH), classified as LRV2 [94].

## 4. Conclusions

Like many neglected diseases, most processes involving leishmaniasis are still not fully optimized, including preventive methods, diagnosis, and treatment, something that constitutes a public health problem in many countries.

VL and CL showed significant differences in all analyses. Latin America led the percentage of reported cases of CL, while countries in the Eastern Mediterranean and Southeast Asia led studies with VL. The HIV coinfection variable was not relevant in studies involving patients with CL. Risk factors associated with TF/CR, in turn, were more diverse for this clinical manifestation, such as drug administration route, host immunology, parasite resistance, the presence of certain clinical and epidemiological conditions, and even the use of different therapeutic regimens (duration, dose, scheme).

TF in VL, on the other hand, was more abundantly related to clinical and immunological factors of the host, with a strong focus on VL/HIV coinfection and therapeutic regimens associated with failure. Regarding the immunological factor, despite a smaller number of associations with CL, this was also pointed out by some authors as responsible for the failure in the treatment. In short, the presence of an immune system prone to failure in manifesting an adequate response was a preponderant element for the occurrence of TF/CR in both the cutaneous and visceral forms, but with greater importance for VL. These observations show the complexity present in the diversity of species that cause leishmaniasis in different geographic areas, highlighting the need to observe specific aspects for each of these clinical manifestations in any investigation carried out.

The works involving experimental research with parasites were conducted mostly with CL species and had a greater focus on changing their sensitivity to drugs and in gene assays. Several genes were observed to be associated with resistance to different drugs, especially antimonials. Some authors point to the possible adaptation of more virulent and infective parasites with increased metacyclogenesis, a situation that may worsen in the future.

We observed studies that identified TF even in patients who resorted to combination therapy, although this phenomenon occurs more frequently in monotherapy. We emphasize here the great association observed between TF and the use of drugs that have been extensively used for years in different endemic regions of the world, such as antimonials, highlighting the need for investment in research that seeks to develop new therapies for leishmaniasis.

We conclude that the phenomenon of TF/CR in leishmaniasis is multifactorial and presents intrinsic peculiarities to each type of clinical manifestation. We found evidence of the existence of risk factors associated with both the host and the etiological agent of the disease and, especially in the case of CL, even with the route of administration of the drug used.

## Figures and Tables

**Table 1:** Data collected from articles categorized as “Experimental with a focus on the parasite”.

References	Article Origin	*Leishmania* Species	Clinical Form	Drugs	Reference Strains and/or Clinical Isolates	Pro	Ama	Experimental Model Used	Possible Mechanisms for Parasites Sensitivity Alterations
[49]	Peru	*L. peruviana*, *L. guyanensis*, *L. braziliensis*, *L. lainsoni e L. mexicana*	CL	Sb^V^	RS/CI	Yes	Yes	Molecular parasite identification/characterization Molecular parasite identification/characterization	Natural interspecific variation
[50]	Sudan	*L. donovani*	VL	Sb^V^	RS/CI	Yes	Yes	*In vitro/in vivo* sensitivity testing and molecular parasite identification/characterization	Factors associated with altered DNA or gene expression level
[51]	Iran	*L. major*	CL	Sb^V^	CI	No	No	Molecular parasite identification/characterization	Factors associated with altered DNA or gene expression level
[52]	Venezuela	*L. amazonensis*	CL	GLIB	RS/CI	Yes	No	Functional assay	Factors associated with altered DNA or gene expression level, altered biochemical pathways, and Presence of virus infection
[53]	Brazil	*L. braziliensis e L. major*	CL	MIL	RS/CI	Yes	Yes	*In vitro/in vivo* sensitivity assays, parasite gene assays, and functional assays	Factors associated with altered DNA or gene expression level
[54]	Brazil	*L. amazonensis*	CL	Sb^III^	RS/CI	Yes	No	*In vitro/in vivo* sensitivity assay and gene assays on the parasite	Factors associated with altered DNA or gene expression level
[55]	Venezuela	*L. mexicana*, *L. amazonensis*, *L. major*, *L. brasiliensis e L. guyanensis*	DCL	GLIB	RS/CI	Yes	No	Gene assays in the parasite and molecular parasite identification/characterization	Factors associated with altered DNA or gene expression level
[56]	Venezuela	*L. mexicana*	DCL	GLIB	RS/CI	Yes	Yes	Parasite gene assays and functional assays	Factors associated with alterations in DNA or gene expression level, factors associated with virulence, and alterations in biochemical pathways
[57]	United States	*L. major*	CL	MIL	RS/CI	Yes	Yes	*In vitro/in vivo* sensitivity testing, gene assays on the parasite, and molecular parasite identification/characterization	Factors associated with changes in DNA or gene expression level and changes in the rate of metacyclogenesis
[58]	Nepal and Belgium	*L. donovani*	VL	Sb^V^	RS/CI	Yes	Yes	*In vitro/in vivo* sensitivity testing and molecular parasite identification/characterization	Altered immune-response-mediation and virulence-associated factors
[59]	Nepal, India and Belgium	*L. donovani*	VL	Sb^V^	CI	Yes	Yes	Gene assays in the parasite and molecular parasite identification/characterization	Changes in the rate of metacyclogenesis
[60]	India	*L. donovani*	VL and PKDL	MIL	RS/CI	Yes	Yes	*In vitro/in vivo* sensitivity testing, parasite gene assays, functional assays, and molecular parasite identification/characterization	Factors associated with virulence, alterations in biochemical pathways and alterations in the rate of metacyclogenesis
[61]	Colombia	*L. braziliensis e L. panamensis*	CL	MIL	RS/CI	Yes	Yes	*In vitro/in vivo* sensitivity testing, gene assays on the parasite, and molecular parasite identification/characterization	Factors associated with altered DNA or gene expression level
[62]	Iran	*L. tropica e L. major*	CL	Sb^V^	CI	Yes	Yes	*In vitro/in vivo* sensitivity testing, gene assays on the parasite, and molecular parasite identification/characterization	Changes in biochemical pathways
[63]	India	*L. donovani*	VL	AmB	CI	Yes	Yes	*In vitro/in vivo* sensitivity testing, parasite gene assays, functional assays, and molecular parasite identification/characterization	Factors associated with altered DNA or gene expression level and altered biochemical pathways
[64]	Índia	*L. donovani e L. amazonensis*	VL	Sb^V^	CI	Yes	Yes	*In vitro/in vivo* sensitivity assay and gene assays on the parasite	Factors associated with altered DNA or gene expression level and altered biochemical pathways
[65]	Índia	*L. donovani*	VL	Sb^V^	CI	Yes	Yes	*In vitro/in vivo* sensitivity testing and molecular parasite identification/characterization	Specific stage response variation
[66]	Algeria, Tunisia and France	*L. infantum*, *L. major e L. killicki*	CL and VL	Sb^V^	CI	Yes	Yes	*In vitro/in vivo* sensitivity testing, gene assays on the parasite and molecular parasite identification/characterization	Factors associated with altered DNA or gene expression level
[67]	Brazil	*L. infantum*	VL	MIL	RS/CI	Yes	No	Gene assays in the parasite	Factors associated with altered DNA or gene expression level

PRO: promastigotes; AMA: amastigotes; CL: cutaneous leishmaniasis; VL: visceral leishmaniasis; DCL: diffuse cutaneous leishmaniasis; PKDL: post-kala-azar dermal leishmaniasis; Sb^V^: pentavalent antimony; Sb^III^: trivalent antimony; GLI: glibenclamide; MIL: miltefosine; AMB: amphotericin B; RS: reference strain; CI: clinical isolate.

**Table 2:** Data collected from articles categorized as “Host-focused population studies”.

References	Article Origin	*Leishmania* Species	Clinical Form	Drugs	Number of Patients	Age Group	Sex	Treatment Failure	Clinical Relapse	HIV Coinfection	Study Methodology	Risk Factors Associated with TF/CR
[70]	Brazil	Not specified	CL	GLU	151	01–69	M: 131 F: 20	Yes (47%)	No	N.I.	Retrospective cohort study	Clinical
[71]	Colombia	*L. panamensis*, *L. braziliensis* and *L. guyanensis*	CL	GLU and MIL	230	02–60	M: 135 F: 95	Yes (15.65%)	No	N.I.	Retrospective cohort study	Clinicians and therapeutic regimen
[72]	Peru	*L. peruviana*, *L. braziliensis* and *L. guyanensis*	CL	SS	119	07–33	M: 73 F: 46	Yes (24.4%)	No	N.I.	Case control	Clinical and epidemiological
[73]	Bolivia	N.I.	CL	Antimonial	30	N.I.	N.I.	Yes (33.33%)	No	N.I.	Immunological investigation	Immunological
[74]	Brazil	N.I.	MCL	GLU and PENT	16	21–80	M: 11 F: 5	No	Yes (50%)	No	Prospective cohort study/immunological investigation	Immunological
[75]	Peru	*L. braziliensis* and *L. guyanensis*	CL	SS	64	mean = 20.5	M: 64 F: 0	Yes (51.6%)	No	No	Retrospective cohort study	Route of drug administration
[76]	Portugal	N.I.	VL	L-AmB and PENT	23	27–48	M: 21 F: 2	No	Yes (26.1%)	VL/HIV	Retrospective cohort study/immunological investigation	Immunological and therapeutic regimens
[77]	Brazil	N.I.	VL	AmB, L-AmB and Antimonials	571	0–60+	M: 362 F: 209	No	Yes (6.8%)	VL/HIV	Retrospective cohort study/immunological investigation	Clinical and immunological
[45]	India and Nepal	N.I.	VL	MIL	853	02–25+	M: 525 F: 328	No	Yes (6.2%)	No	Prospective cohort study	Clinical and immunological
[78]	Sudan	N.I.	VL	SS	25	N.I.	N.I.	Yes (20%)	No	No	Cross-section/immunological investigation	Immunological
[79]	Nepal	N.I.	VL	MIL	120	N.I.	M: 74 F: 46	No	Yes (20.0%)	No	Prospective cohort study	Clinical
[80]	Ethiopia	N.I.	VL	L-AmB, SS, L-AmB + MIL, SS + PAR, PENT	12	05–41	M: 12 F: 0	No	Yes (100%)	VL/HIV	Case series	Immunological
[81]	India	N.I.	PKDL and VL	L-AmB, MIL + PAR and L-AmB + MIL	1761	02–80	M: 1.067 F: 694	No	Yes (4.5%)	N.I.	Clinical trial	Clinical, immunological, and therapeutic regimens
[82]	Spain	N.I.	VL	GLU, AmB, L-AmB and MIL	4	32–45	M: 3 F: 1	No	Yes (75%)	VL/HIV	Retrospective cohort study	Therapeutic regimens
[83]	Spain	N.I.	VL	GLU, AmB, PENT, L-AmB	155	30–37	M: 129 F: 26	No	Yes (24%)	VL/HIV	Retrospective cohort study/immunological investigation	Clinical, immunological, and therapeutic regimens
[84]	Spain	N.I.	VL	GLU and AmB	10	31–38	M: 6 F: 4	No	Yes (70%)	VL/HIV	Prospective cohort study /immunological investigation	Immunological
[85]	French Guiana	*L. guyanensis*	CL	PENT and GLU	75	Not specified	M: 66 F: 9	Yes (5%)	Yes (17.33%)	No	Prospective cohort study /immunological investigation	Presence of LRV virus
[86]	Kenya	*L. tropica*	CL	SS	52	05–52	M: 21 F: 31	Yes	Yes (44.2%)	N.I.	Epidemiological	Therapeutic regimens
[87]	French Guiana and mainland France	*L. guyanensis*	CL	PENT	73	21–44	M: 72 F: 1	Yes (32.8%)	No	N.I.	Case series	Route of drug administration

Drugs and therapies separated by “and” indicate different monotherapy regimens, while drugs joined by “+” indicate combination therapy. M: male; F: female; CL: cutaneous leishmaniasis; VL: visceral leishmaniasis; MCL: mucocutaneous leishmaniasis; PKDL: post-kala-azar dermal leishmaniasis; GLU: meglumine antimoniate (Glucantime^®^); SS: sodium stiboglucanate (Pentostan^®^); PENT: pentamidine; PAR: paromomycin; AmB: amphotericin B; L-AmB: liposomal amphotericin B; MIL: miltefosine; N.I.: not informed.

## Data Availability

The datasets generated during and/or analyzed during the current study are available from the corresponding author on reasonable request.

## Supplementary Materials

The following supporting information can be downloaded at: https://www.mdpi.com/article/10.3390/tropicalmed8090430/s1. Table S1: Summary of articles categorized as case reports.
