# Supplementary material for: Systematic Review of Treatment Failure and Clinical Relapses in Leishmaniasis from a Multifactorial Perspective: Clinical Aspects, Factors Associated with the Parasite and Host
Source: Trop Med Infect Dis. 2023 Aug 29;8(9):430. doi: 10.3390/tropicalmed8090430 (PMC10534360; doi:10.3390/tropicalmed8090430)
Supplement: Supplementary file 1 [file tropicalmed-08-00430-s001.zip › tropicalmed-2492789-supplementary.pdf]

Table S1: Summary of articles categorized as case reports

References	Place of occurrence	Gender	Age	Clinical manifestation	Leishmania species	Unsuccessful treatment	Successful treatment	Clinical outcome	Immuno-suppression and comorbidities	Suggested mechanisms for failure/relapse by authors
[12]	Venezuela	M	58	CL	*L. braziliensis*	AmB e FLU	L-AmB	Clinical cure	N.I.	Dose-dependent resistance of the species
[13]	Brazil	M	18	CL	*L. braziliensis*	GLU	GLU + Pentoxifylline	Clinical cure	No	Inappropriate initial treatment
[14]	Brazil	M	3	VL	*L. infantum*	L-AmB	AmB+GLU+SS	Clinical cure	N.I.	N.I.
[15]*	French Guiana	M (7 patients)	N.I.	CL	*L. guyanensis*	PENT (IL or IM)	PENT	Clinical cure	N.I.	Route of drug administration
[16]	Belgium	F	52	VL, CL e MCL	*L. infantum*	L-AmB e AmB lipid complex. MIL e AmB.	GLU (CL) and PAR + MIL (VL)	Death	Yes	Host immunological factors
[17]	Greece	F	64	CL	*L. tropica*	GLU	GLU+ cryosurgery with liquid nitrogen	Clinical cure	N.I.	N.I.
[18]*	Brazil	M/M (2 patients)	23/ 54	VL	*L. infantum*	GLU, AmB e PENT/ GLU e AmB	Splenectomy + AmB/ Splenectomy + AmB	Clinical cure	No	N.I.
[19]*	Colombia	F/M/M/ M/M (5 patients)	0.6/5/ 23/27/25	CL	*L. panamensis*	GLU IL/ GLU IL/ GLU IL/ GLU IL, GLU IM	GLU IM/ GLU-IM/ GLU IM/ GLU-IM/ Cryotherapy	Clinical cure	N.I.	N.I.
[20]	France	F	8	VL	*L. infantum*	L-AmB e AmB	Anti-TNFα	Clinical cure	Yes	Host immunological factors
[21]	Greece	M	64	VL	*L. infantum*	GLU	L-AmB	Clinical cure	No	Mixed infection by two strains
[22]	Honduras	M	3	VL	N.I.	GLU	Not applicable	Death	No	Inappropriate initial treatment
[23]	Pakistan	M	50	CL	*L. tropica*	SS e AmB	SS+ Itraconazole	Clinical cure	No	N.I.
[24]	Spain	F	87	CL	N.I.	Rifampicin and PAR cream 15%	GLU IL	Clinical cure	No	Inappropriate initial treatment
[25]	Brazil	M	32	CL	*L. naiffi*	PENT	Surgical removal of the lesion	Clinical cure	No	Presence of LRV virus
[26]	Argentina	M	65	MCL	*L. braziliensis*	AmB e MIL	Itraconazole + GLU IL+PENT IV+L-AmB aerosol	Clinical cure	Yes	Host immunological factors
[27]*	France/Algeria	M/F	44/33 (2 patients)	VL	*L. infantum*	GLU, AmB IV, GLU/L-AmB	PENT	Clinical cure/N.I.	Yes/Yes	Host immunological factors/Host immunological factors
[28]	Syria	M	8	CL	N.I.	GLU IL and GLU IM	FLU + cryotherapy	Clinical cure	No	Host immunological factors
[29]	Palestine	M	12	CL	*L. tropica*	SS IL	Not applicable	N.I.	No	Parasite resistance or treatment interruption

References	Place of occurrence	Gender	Age	Clinical manifestation	Leishmania species	Unsuccessful treatment	Successful treatment	Clinical outcome	Immuno-suppression and comorbidities	Suggested mechanisms for failure/relapse by authors
[30]	Asia	M	80	VL	*L. donovani*	AmB-L	Not applicable	Death	Yes	Host immunological factors
[31]	India	M	43	VL	*L. donovani*	MIL, L-AmB and AmB	L-AmB + MIL	Clinical cure	Yes	Host immunological factors
[32]	Albania	F	8	VL	N.I.	GLU	L-AmB	Clinical cure	Yes	N.I.
[33]	Brazil	M	0.6	VL	N.I.	GLU, AmB and L-AmB	GLU	Clinical cure	No	N.I.
[34]*	Colombia	F/M (2 patients)	1.5/3	VL	*L. infantum*	GLU and PENT	L-AmB	Clinical cure	No	N.I.
[35]	Nepal	M	19	VL	*L. donovani*	MIL	AmB	Clinical cure	No	Parasite resistance
[36]	Brazil	M	46	DCL	*L. amazonensis*	GLU, L-AmB, GLU, L-AmB+PENT, PENT, L-AmB+GLU, GLU	Not applicable	N.I.	Yes	Host immunological factors
[37]*	France/Georgia /Georgia/ Algeria	M/ F/M/M (4 patients)	58/1.5/ 37/75	VL	N.I./ N.I./N.I./ *L. infantum*	L-AmB, L-AmB+MIL/ L-AmB, L-AmB+MIL/ L-AmB and GLU/ L-AmB	GLU/ GLU/ GLU and MIL/ GLU	Clinical cure	Yes/ No/ Yes/ Yes	Host immunological factors/ N.I./ Host immunological factors/ Host immunological factors

* These articles describe case reports for more than one patient. The information for these patients in the columns are separated by slashes (/).
In the columns "successful treatment" and "unsuccessful treatment", drugs and therapies linked by "and" indicate treatment cycles at different times, while drugs linked by "+" indicate combined therapy
M-male; F-female; CL- cutaneous leishmaniasis; VL- visceral leishmaniasis; MCL- mucocutaneous leishmaniasis; DCL- diffuse cutaneous leishmaniasis; FLU- fluconazole; GLU- meglumine antimoniate (Glucantime®) ; SS- sodium stiboglucanate (Pentostan®); PENT- pentamidine; PAR- paromomycin; AmB- amphotericin B; L-AmB- liposomal amphotericin B; MIL- miltefosine; IL- intralesional; IM- intramuscular; N.I.- not informed.
